# Supplementary material for: Prototype or Exemplar Representations in the 5/5 Category Learning Task
Source: Behav Sci (Basel). 2024 May 31;14(6):470. doi: 10.3390/bs14060470 (PMC11200643; doi:10.3390/bs14060470)
Supplement: Supplementary file 1 [file behavsci-14-00470-s001.zip › behavsci-2984824-supplementary/introduce.pdf]

Introduction

GCM

1， Put in comparing exemplar. A category in typea.txt;B categroy in typeb.txt。
2， Put in other comparing exemplar. A and B together in typeall.txt。
3， Put in data from experiment in expdata.txt.（percentage of A responds）
4， Run run.m。

MPM

1， Put in comparing exemplar (prototype). A category prototype in typea.txt;B category prototype in typeb.txt。
2， Put in other comparing exemplar. A and B together in typeall.txt。
3， Put in data from experiment in expdata.txt.（percentage of A responds）
4， Run run.m。

Outcome:

5 parameters, w1,w2,w3,w4 and c

Fval means fitness of the model, SSD
